# MicroRNA-503 Exacerbates Myocardial Ischemia/Reperfusion Injury via Inhibiting PI3K/Akt- and STAT3-Dependent Prosurvival Signaling Pathways

**DOI:** 10.1155/2022/3449739

**Published:** 2022-05-17

**Authors:** Yanjing He, Yin Cai, Tianhao Sun, Liangqing Zhang, Michael G. Irwin, Aimin Xu, Zhengyuan Xia

**Affiliations:** ^1^State Key Laboratory of Pharmaceutical Biotechnology, Department of Medicine, The University of Hong Kong, Hong Kong; ^2^Department of Anaesthesiology, The University of Hong Kong, Hong Kong; ^3^Department of Health Technology and Informatics, The Hong Kong Polytechnic University, Hong Kong; ^4^Department of Orthopaedics and Traumatology, The University of Hong Kong-Shenzhen Hospital, Shenzhen, China; ^5^Department of Anaesthesiology, Affiliated Hospital of Guangdong Medical University, Zhanjiang, China

## Abstract

Acute myocardial infarction is a leading cause of death worldwide, while restoration of blood flow to previously ischemic myocardium may lead to ischemia/reperfusion (I/R) injury. Accumulated evidence shows that microRNAs play important roles in cardiovascular diseases. However, the potential role of microRNA-503 (miR-503) in myocardial I/R injury is little known. Thus, this study is aimed at determining whether and how miR-503 affects myocardial I/R injury *in vivo* and *in vitro*. A mouse model of myocardial I/R injury and H9c2 cell model of hypoxia/reoxygenation (H/R) injury were established. The postischemic cardiac miR-503 was downregulated *in vivo* and *in vitro*. Mechanistically, PI3K p85 and Bcl-2 are miR-503 targets. The post-ischemic cardiac PI3K p85 protein level was decreased *in vivo*. Agomir-503 treatment exacerbated H/R-induced injuries manifested as decreased cell viability, increased lactate dehydrogenase activity, and cell apoptosis. Agomir-503 treatment reduced cell viability under normoxia as well and reduced both PI3K p85 and Bcl-2 protein levels under either normoxia or H/R condition. It reduced phosphorylation of Stat3 (p-Stat3-Y705) and Akt (T450) in cells subjected to H/R. In contrast, Antagomir-503 treatment attenuated H/R injury and increased p-Stat3 (Y705) under normoxia and increased p-Akt (T450) under either normoxia or H/R condition. It is concluded that miR-503 exacerbated I/R injury via inactivation of PI3K/Akt and STAT3 pathways and may become a therapeutic target in preventing myocardial I/R injury.

## 1. Introduction

Acute myocardial infarction (MI) is a leading cause of death worldwide [1]. Prompt restoration of coronary blood flow with either thrombolytic therapy or primary percutaneous coronary intervention (PPCI) is the most effective way of reducing myocardial infarction size and improving the prognosis; however, the process of reperfusion itself can paradoxically induce injury; this phenomenon is termed myocardial ischemia/reperfusion (I/R) injury [2]. Several critical factors, including oxidative stress, intracellular and mitochondrial calcium overload, rapid intracellular pH correction, mitochondrial permeability transition pore opening, and inflammation, are driving the reperfusion injury [3]. Previous studies have shown that three major intrinsic prosurvival pathways are activated at the time of reperfusion, that is, reperfusion injury salvage kinase pathway (including phosphoinositide 3-kinase (PI3K)/Akt and MEK1/2-ERK1/2 pathways), the survivor activator factor enhancement pathway (namely, Janus kinase/signal transducer and activation of transcription 3 (JAK-STAT3) pathway), and the cGMP/PKG pathway [4, 5]. The prosurvival pathways protect against I/R injury through mediating the activation of downstream mediators, such as endothelial nitric oxide synthase (eNOS), glycogen synthase kinase-3*β*, and the mitochondrial ATP-dependent potassium channel, exhibiting in delaying MPTP opening, decreasing reactive oxygen species generation and cell apoptosis [6–8]. Although therapeutic intervention for patients receiving PPCI has been optimized and the mortality following MI is reduced, the rate of heart failure in patients who survived MI and PPCI is increasing, bringing heavy economic burden to the society [6, 9]. Therefore, a novel therapy for alleviating myocardial I/R injury is still an unmet need.

The mammalian phosphoinositide 3-kinases (PI3Ks) family includes three classes members, that is Class I, Class II, and Class III; Class I PI3Ks is composed of catalytic subunits and regulatory subunits; P13K p85 is one of the regulatory subunits [10]. The protein kinase Akt family includes Akt1, Akt2, and Akt3; mainly, the former two are expressed in the adult heart [11]. Akt1 can be phosphorylated at its Serine124, Threonine308 (T308), Threonine450 (T450), and Serine473 (S473) residues. And Akt2 can be phosphorylated at its Threonine309 and Serine474 residues [12]. T308 and S473 are the major phosphorylated residues in Akt1, and phosphorylation of both residues generates maximal Akt1 activity [13]. Akt phosphorylation on T450 is important in regulating the reactivation of Akt after hypoxia or ischemia injury and the ensuing reoxygenation or reperfusion [14]. Therefore, we detected Akt1, Akt2, and p-Akt (T450 and T308) in this study.

The mammalian JAKs family is composed of Jak1, Jak2, Jak3, and Tyk2 [15]. Activation of Jak1 and Jak2 results in Stat3 activation by tyrosine phosphorylation [7, 16]. Stat3, as a transcription factor, is phosphorylated on tyrosine residue (Y705) in the cytoplasm and translocated to the nucleus, where it is further phosphorylated on its serine residue (S727), binding to DNA and leading to target gene transcription, such as B cell lymphoma protein 2 (Bcl-2) and Bcl-2-related protein long form of Bcl-x (Bcl-XL) [7, 17]. As the JAK/STAT3 signaling pathway is an important prosurvival signaling pathway in myocardial I/R injury, so we also explored the alterations of related proteins in this pathway.

MicroRNAs (miRs) are small noncoding RNAs with around 22 nucleotides in length, which regulate target genes via translational repression or mRNA degradation [18]. miRs are involved in various biological processes, such as cell differentiation, proliferation, apoptosis, metabolism, and development [19, 20]. Numerous miRs are implicated in cardiovascular diseases, such as miR-15 [21] in cardiac injury, miR-25 in heart failure [22], miR-29 in aortic dilation [23], and miR-133 in cardiac hypertrophy [24]. miR-503 is an intragenic miR clustered with miR-424 and located on chromosome Xq26.3 in human and clustered with miR-322 in rodents [20]. Accumulated evidence has shown that miR-503 is implicated in human diseases, such as diabetes mellitus [25], pulmonary arterial hypertension [26], and cancer [27, 28]. In an ex vivo rat model of myocardial I/R injury, microarray analysis showed that miR-503 was downregulated in the anterior wall of left ventricles of rats exposed to 30 min of ischemia and 120 min of reperfusion [29]; however, little is known about the role of miR-503 in myocardial I/R injury. Therefore, the aim of this study is to investigate whether and how miR-503 regulates myocardial I/R injury.

In this study, we first investigated whether miR-503 was dysregulated in a mouse model of myocardial I/R injury or H9c2 cells exposed to hypoxia/reoxygenation (H/R) injury. We next explored the role of miR-503 in H9c2 cells by overexpression or inhibition of miR-503 under either normoxia or H/R condition. As PI3K p85 and Bcl2 are the predicted targets genes of miR-503, we have detected protein PI3K p85, Bcl-2, and other related proteins involved in the P13K/Akt and JAK/STAT3 pathways, such as Akt1, Akt2, p-Akt (T450 and T308), Stat3, and p-Stat3 (Y705) and demonstrated that overexpression of miR-503 was detrimental to cells during H/R injury by inhibiting both the PI3K/Akt and STAT3 signaling pathways.

## 2. Materials and Methods

### 2.1. Reagents

Dulbecco's modified Eagle's medium (DMEM), opti-MEM, fatal bovine serum (FBS), 0.25% Trypsin-EDTA, and penicillin/streptomycin were ordered from GIBCO, Thermo Fisher Scientific (Waltham, MA, USA). The primers of miR-503 and U6 snRNA, the miR-503 agomir, antagomir, and their corresponding negative control (NC) were ordered from RiboBio (Guangzhou, China). The rabbit polyclonal anti-Bcl-2 antibody (ab196495) was purchased from Abcam (Cambridge, UK). The anti-PI3 Kinasep85 (4292S), anti-phospho-Akt (T450) (9267S), anti-phospho-Akt (T308) (4056S), anti-JAK2 (3230S), anti-phospho-JAK2 (Y1007/1008) (3771S), anti-phospho-Stat3(Y705) (9131S), anti-Stat3 (9139S), anti-Akt1 (2938S), anti-Akt2 (3063S), anti-Bax(2772S), anti-*β*-actin (4967S), and anti-GADPH (2118S) antibodies were purchased from Cell Signaling Technology (CST) (Danvers, MA, USA). All other analytical grade chemical reagents were purchased from standard commercial companies.

### 2.2. Mouse Model of Myocardial I/R Injury

This study was approved by the Committee on the Use of Live Animals in Teaching and Research of The University of Hong Kong (CULATR no. 4169-16). Male adult C57BL/6N mice (12 weeks of age) were ordered from and housed in the Laboratory Animal Unit (The University of Hong Kong). All mice were housed on a 12 hours (h) light/dark cycle in a temperature-controlled room (25 ± 2°C) with free access to water and food. Mice were randomly divided into the sham and I/R groups (*n* = 12 mice per group) and received sham operation or myocardial I/R operation after anesthesia. According to the needs of different experiments, each group were separated into subgroups. The surgical procedures were performed as previous protocols [30, 31]. The left anterior descending coronary artery of the mouse heart was ligated with a 7-0 silk suture for 30 minutes (min) followed by 2 h of reperfusion. Sham operation was performed by passing a suture under the coronary artery without ligation. Animals were sacrificed at the end of 2 h of reperfusion. Myocardial infarct size (IS) was determined by using 5%Evans blue/1%TTC (2, 3, 5-triphenyltetrazolium chloride) staining. The blue area reflects viable area, red area reflects area at risk (AAR), and white area reflects infarct area; IS was expressed as a percent of the AAR. In the subset of animals, after sham or I/R operations, plasma was extracted from the blood and stored at -80°C until use. The whole heart tissues obtained after operation without staining were snap frozen in liquid nitrogen and stored at -80°C until RNA isolation or protein extraction.

### 2.3. Cell Culture

The rat myocardium-derived cell line H9c2 obtained from the American Type Culture Collection (ATCC, Manassas, VA, USA) was cultured in complete growth medium DMEM (1 g/l glucose) supplemented with 10% FBS and 1% penicillin/streptomycin (100 U/ml penicillin and 100 *μ*g/ml streptomycin) and incubated in 5% CO_2_ in air at 37°C (Thermo Scientific, Waltham, MA, USA). To establish simulated I/R model *in vitro*, H9c2 cells were exposed to glucose deprivation and hypoxia for 10 h followed by 6 h of reoxygenation as described [32]. Briefly, H9c2 cells were cultured in DMEM without glucose and FBS and incubated in a modular incubator chamber (Billups-Rothenberg Inc., SanDiego, CA, USA) with <1%O_2_ and 5%CO_2_/95%N_2_ at 37°C for 10 h to simulate ischemia (hypoxia, H). By replacing the medium with complete growth medium saturated with 95%air and 5%CO_2_, the cells were incubated under normoxia at 37°C for 6 h (reoxygenation, R). The cells were cultured in complete growth medium and incubated under normoxia accordingly as the control (Con).

### 2.4. Cell Transfection

After cell seeding for 24 h, H9c2 cells were transfected with Agomir-503, Antagomir-503, or their corresponding NC using Lipofectamine 2000 (Invitrogen, Carlsbad, CA, USA) separately. Serum/glucose deprivation and H/R treatment were used after transfection for 34 h in H/R groups. To overexpress miR-503, cells were divided into the Con+NC, Con+Agomir-503, H/R+NC, and HR+Agomir-503 groups randomly. To inhibit miR-503, cells were divided into Con, H/R, H/R+NC, and H/R+Antagomir-503 groups. The cells were tested mycoplasma negative before transfection.

### 2.5. MTT Assay

H9c2 cells were cultured in clear bottom 96-well plates, and cell viability was determined by Thiazolyl Blue Tetrazolium Bromide (MTT) assay [33] according to the manufacturer's instructions. After cell transfection and H/R stimulation, the culture medium was removed from wells, and 50 *μ*l MTT solution (0.8 mg/ml) (Sigma, St Louis, USA) was added into each test well. The plates were incubated with MTT solution at 37°C incubator for 2 h. Then, after removing the MTT solution and the addition of 100 *μ*l isopropanol to each well, the plate was shaken for 15 min at room temperature until the blue dots totally dissolved to form a homogeneous blue solution. The absorbance was measured by a microplate reader (BioTek, Vermont, USA) at wavelength of 570 nm; cell viability was calculated by OD sample/OD control × 100%.

### 2.6. Detection of Lactate Dehydrogenase

The cytoplasmic enzyme lactate dehydrogenase (LDH) is released into the cell culture medium from the damaged cells. LDH activity was determined in cell culture supernatant with the use of Cytotoxicity Detection Kit (Roche, Mannheim, Germany) according to the manufacturer's instructions as described [34]. The absorbance was measured at wavelength of 492 nm with reference wavelength 620 nm. The OD value was normalized by cell protein amount.

### 2.7. Detection of CK-MB

Creatine Kinase MB Isoenzyme (CK-MB) is a specific marker of cardiac injury. CK-MB in the serum was determined using enzyme-linked immunosorbent assay kit according to the manufacturer's instructions (Cloud-Clone Corp., TX, USA) as described [35]. Briefly, standards or samples were added to wells which were precoated with an antibody specific to CK-MB and incubated at 37°C for 1 h; then, the liquid in each well was discarded, and biotin-conjugated antibody was added and incubated for 1 h at 37°C. Thereafter, each well was washed and incubated with horseradish peroxidase- (HRP-) avidin for 1 h at 37°C. Next, the liquid in each well was removed and washed; then, the TMB substrate was added. The plate was incubated for 10-20 min at 37°C in dark. At last, after adding stop solution, the OD value was read with a microplate reader at wavelength of 450 nm/570 nm immediately.

### 2.8. Reverse Transcription Quantitative Polymerase Chain Reaction

Total RNA was extracted from H9c2 cells or heart tissues using RNAiso Plus reagent according to the manufacturer's instructions (TakaRa, Japan) as described [36]. The whole heart tissue was ground into powder with liquid nitrogen, and about 50 mg powder was homogenized with RNAiso Plus reagent. cDNA was synthesized from 1 *μ*g of total RNA from each sample with PrimeScript RT reagent kit (Takara) and Bulge-Loop™ miRNA RT-qPCR Starter Kit (RiboBio). The expression level of mature miR-503 was quantified by RT-qPCR using SYBR Premix Ex Taq II kit (Takara). U6 was regarded as an internal reference of miR-503. Amplification of cDNA was performed on a StepOnePlus Real-time PCR System (Applied. Biosystems). The reaction was initiated at 95°C for 30 sec, followed by 40 cycles of 95°C for 5 sec and 60°C for 30 sec. The 2^-∆∆CT^ was used as relative quantification method.

### 2.9. Apoptosis Assay

Cell apoptosis was detected using In Situ Cell Death Detection Kit (Roche) according to the manufacturer's instructions as described [31]. In brief, H9c2 cells were seeded in chamber slides, and both negative and positive labeling controls were set. At the end of reoxygenation, the cells were fixed in 4% paraformaldehyde in phosphate-buffered saline, blocked in 3% H_2_O_2_ in methanol, incubated in permeabilization solution, and sequentially applied with TUNEL (terminal deoxynucleotidyl transferase-mediated dUTP nick end labeling) reaction mixture, converter-POD, and DAPI staining. Images were observed and taken by Olympus BX41 fluorescence microscope supplied with a digital camera (Olympus, Tokyo, Japan). When taking images, consistent parameters, such as scale and exposure time, are set for each image in order to get comparable images. The green color in TUNEL staining represents apoptotic cells, while nuclei is blue counterstained by DAPI. At least five images (magnification ×100) were taken randomly for each sample. The number of total cells and TUNEL-positive cells was quantified by ImageJ software. The percentage of apoptotic cells is calculated by normalizing DAPI-positive cells.

### 2.10. Bioinformatics

Potential target genes of miR-503 were obtained from TargetScan Rat v7.2 (http://www.targetscan.org). PIK3R1 (encoding P13K p85, phosphoinositide-3-kinase regulatory subunit 1) and Bcl2 (B cell lymphoma protein 2) are predicted to be the target genes of miR-503.

### 2.11. Western Blotting Analysis

Cells were washed with ice-cold PBS and lysed in cell lysis buffer (CST), supplemented with phosphatase and protein inhibitor Cocktail tablets (Roche). About 50 mg of heart tissue powder was homogenized in lysis buffer by Rotor Stator disperser homogenizer. After centrifugation for 15 min at 13,200 rpm (4°C), the protein concentration was detected by Bradford method (Bio-Rad, CA, USA). Equal amounts of protein were loaded onto 10% or 12.5% SDS-PAGE gels and transferred to PVDF membranes (0.2 *μ*m). The membranes were blocked in 5% *w*/*v* nonfat dry milk, 1 **×** TBS, 0.1% Tween® 20 at room temperature for 1 h and then incubated with diluted primary antibody in 5% *w*/*v* bovine serum albumin (BSA)-TBST at 4°C overnight. The membranes were washed with 0.1% TBST three times and incubated with a HRP-linked secondary antibody for 1 h at room temperature. The membranes were detected by ChemiDocTM Touch Imaging System (BIO-RAD). The protein levels were quantified by ImageJ software and normalized to *β*-actin or GADPH as an internal control.

### 2.12. Statistical Analysis

Data are expressed as mean ± standard error of the mean (SEM). *In vitro* experiments were repeated at least 3 times independently. Statistical analysis was determined by Student's *t*-test, nonparametric Mann–Whitney test between two groups, or one-way ANOVA followed by the Newman-Keuls multiple comparison test for multiple group comparisons (GraphPad Prism version 7). A value of *P* < 0.05 was considered statistically significant.

## 3. Results

### 3.1. The Cardiac Expression Level of miR-503 *In Vivo* and *In Vitro*

**Figure 1:**
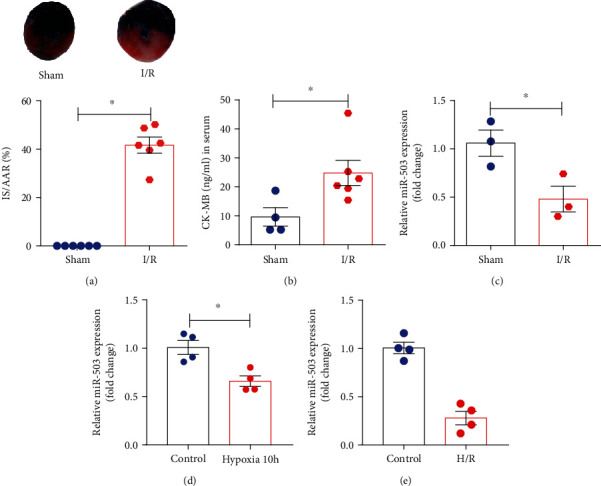
The expression level of miR-503 *in vivo* and *in vitro*. (a) Representative images, IS was expressed as a percentage of the AAR. (b)The CK-MB concentration in serum. (c–e) miR-503 expression in mouse hearts and in H9c2 cells, quantified by RT-qPCR. Data are expressed as mean ± SEM. Student's *t*-test was applied. (a) *n* = 6 mice/group, (b) *n* = 4-6 mice/group, and (c) *n* = 3 mice/group. ^∗^*P* < 0.05 vs. the sham. (d, e) *n* = 4/group. ^∗^*P* < 0.05 vs. the control.

To investigate the role of miR-503 in myocardial I/R injury, we have established the mouse model of myocardial I/R injury and H9c2 cell model of H/R injury. The mice after myocardial I/R injury exhibited myocardial infarction, and serum CK-MB concentration was increased significantly compared with the sham group (Figures 1(a) and 1(b)). RT-qPCR analysis showed that the expression level of miR-503 in heart tissues was decreased significantly in the I/R group compared with the sham group (Figure 1(c)). *In vitro*, results showed that miR-503 was decreased significantly under both hypoxia for 10 h and H/R conditions compared with control (Figures 1(d) and 1(e)). These results indicated that miR-503 was dysregulated in the pathogenesis of myocardial I/R injury.

### 3.2. Overexpression of miR-503 Exacerbated Cell Injury Induced by H/R

**Figure 2:**
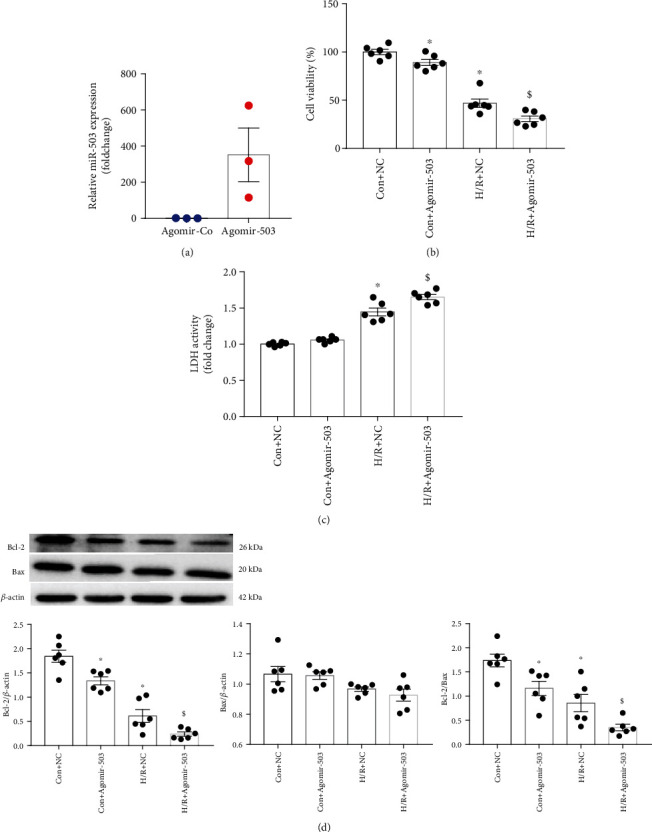
The effects of miR-503 overexpression on H9c2 cell H/R injury. (a) miR-503 expression level after transfection of Agomir-503 under normoxia, quantified by RT-qPCR. Agomir-Co: negative control of Agomir-503. (b) Cell viability. (c) LDH activity. (d) Western blotting analysis of Bcl-2 and Bax and densitometry analysis of proteins. Data are expressed as mean ± SEM. (a) *n* = 3/group, ^∗^*P* < 0.05 vs. Agomir-Co. Student's *t*-test was applied. (b–d) *n* = 6/group. ^∗^*P* < 0.05 vs. Con+NC; ^$^*P* < 0.05 vs. H/R+NC. NC: negative control of Agomir-503. Con indicates control. One-way ANOVA followed by Newman-Keuls multiple comparison test was applied.

The expression level of miR-503 was downregulated *in vivo* and *in vitro*; thus, we hypothesized that upregulation of miR-503 *in vitro* might alleviate cell injury upon H/R stimulation. Agomir-503 was applied to overexpress miR-503. We detected the miR-503 expression level by RT-qPCR to confirm the transfection efficiency. RT-qPCR analysis showed that miR-503 expression level was increased significantly in cells by Agomir-503 treatment under normoxia (Figure 2(a)). The cell viability was decreased significantly by Agomir-503 treatment under normoxia, as detected by MTT assay (Figure 2(b)), and it was also decreased significantly in the H/R+negative control of Agomir-503 (NC) group compared with the Con+NC group and further decreased by Agomir-503 treatment (Figure 2(b)). The LDH activity in cell culture supernatant was significantly increased in the H/R+NC group compared with the Con+NC group and further increased by Agomir-503 (Figure 2(c)). Western blotting analysis showed that the antiapoptotic protein Bcl-2 level was decreased significantly by Agomir-503 treatment under normoxia; it was also significantly decreased after H/R stimulation and further decreased by Agomir-503 treatment. For proapoptotic protein Bcl2-associated X protein (Bax), there was no significance among groups (Figure 2(d)). The ratio of Bcl-2 to Bax was decreased significantly under H/R condition, and it was also decreased by Agomir-503 treatment under either normoxia or H/R condition (Figure 2(d)).

**Figure 3:**
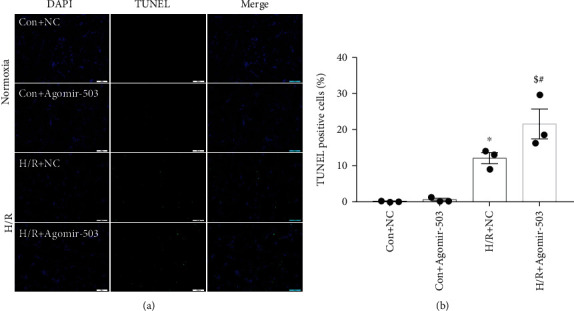
miR-503 overexpression in H9c2 cells increased posthypoxic cell apoptosis. (a) Representative TUNEL-stained images under both normoxia and H/R conditions. TUNEL staining was green color representing apoptotic cells, nuclei counterstained by DAPI showed blue. Scale bar = 100 *μ*m. (b) Quantification of TUNEL-positive cells by ImageJ software. The percentage of TUNEL-positive cells was calculated by normalizing DAPI-positive cells. Data are expressed as mean ± SEM. *n* = 3/group. ^∗^*P* < 0.05 vs. Con+NC, ^$^*P* < 0.05 vs. H/R+NC, ^#^*P* vs. Con+Agomir-503. One-way ANOVA followed by Newman-Keuls multiple comparison test was applied.

In addition, apoptotic cells were detected by TUNEL assay.  Figure 3(a) showed the representative TUNEL staining images under both normoxia and H/R conditions, and Agomir-503 treatment did not cause obvious apoptotic cell death under normoxia, but the percentage of apoptotic cells was significantly increased in the H/R+NC group compared with the Con+NC group, and it was increased further by Agomir-503 treatment, as quantified in Figure 3(b). Contrary to our expectation, these findings indicated that upregulation of miR-503 exacerbated the detrimental effects of H/R in H9c2 cells.

### 3.3. miR-503 Exacerbated H/R Induced Cell Injury via Inactivating PI3K/Akt and STAT3 Signaling Pathways

The molecular mechanism underlying the effects of miR-503 in myocardial I/R injury is unclear. PI3K/Akt and JAK/STAT3 signaling pathways are important prosurvival signaling pathways in myocardial I/R injury; we investigated whether miR-503 regulated the myocardial I/R injury via these pathways.

**Figure 4:**
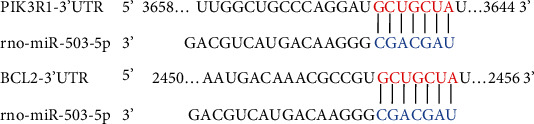
Predicted target genes PIK3R1 and Bcl2 of miR-503. Predicted duplex formation between miR-503 and rat PIK3R1 or Bcl2.

**Figure 5:**
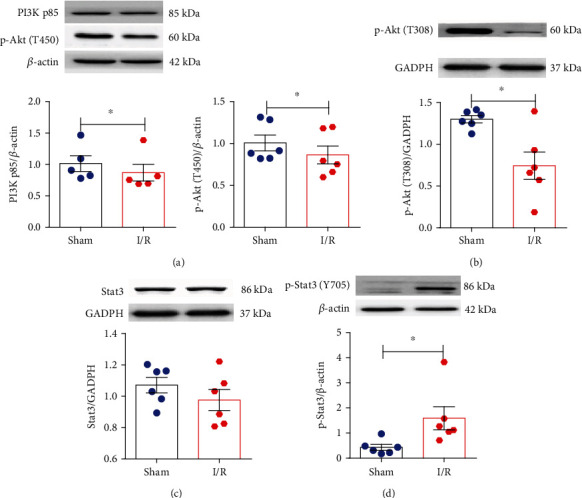
The protein expression levels of PI3K p85, p-Akt (T450 and T308), Stat3, and p-Stat3 in mouse heart tissues. (a–d) Western blotting analysis of PI3K p85, p-Akt (T450 and T308), Stat3, and p-Stat3 (Y705) and densitometry analysis of proteins. Data are expressed as mean ± SEM, *n* = 5-6 mice/group. ^∗^*P* < 0.05 vs. the sham. (a, c, d) Student's *t*-test was applied. (b) Nonparametric Mann–Whitney test was applied.

**Figure 6:**
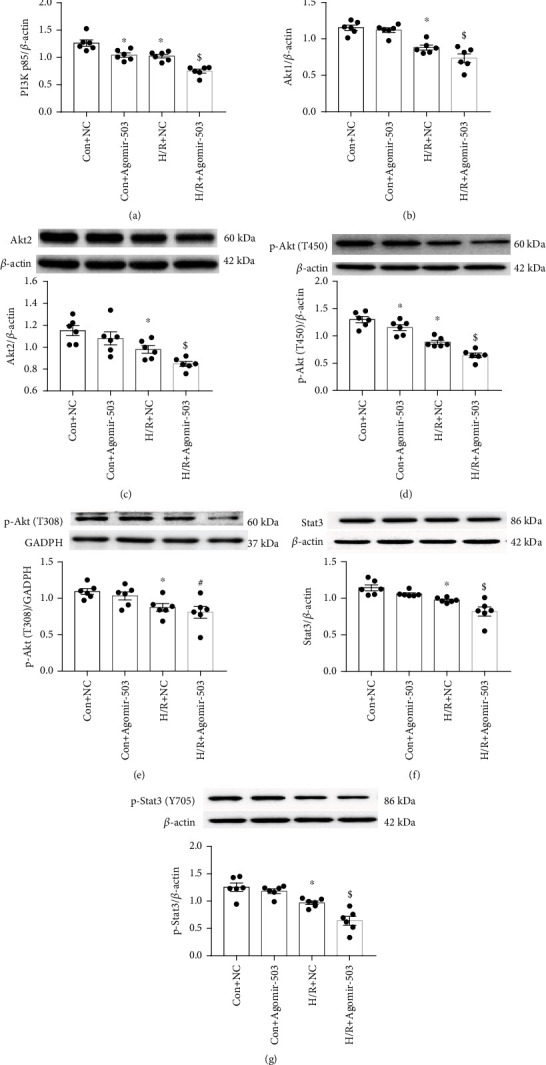
miR-503 overexpression inhibited the activation of PI3K/Akt and STAT3 pathways *in vitro*. (a–g) Western blotting analysis of PI3K p85, Akt1, Akt2, p-Akt (T450 and T308), Stat3, and p-Stat3 (Y705) and densitometry analysis of proteins. Data are expressed as mean ± SEM, *n* = 6/group. (a–g) ^∗^*P* < 0.05 vs. Con+NC, ^$^*P* < 0.05 vs. H/R+NC, ^#^*P* < 0.05 vs. Con+Agomir-503. One-way ANOVA followed by Newman-Keuls multiple comparison test was applied.

With TargetScan bioinformatics software, PIK3R1 (encoding PI3K p85) and Bcl2 are the predicted target genes of miR-503 (Figure 4). First, we detected protein expression levels of PI3Kp85 and its downstream protein phosphorylated Akt (also named protein kinase B) (p-Akt, T450, or T308) *in vivo*. Stat3 and phosphorylated Stat3 (p-Stat3, Y705) protein were also detected. It showed that PI3K p85 protein level was decreased in mouse heart tissues after I/R, and both p-Akt (T450) and p-Akt (T308) expression levels were also decreased (Figures 5(a) and 5(b)). In contrast, the level of p-Stat3 was upregulated in the I/R group, while total Stat3 did not significantly differ between groups (Figures 5(c) and 5(d)). Then, we further examined the impact of miR-503 on the related proteins *in vitro*. Consistent with *in vivo* experiments, the results demonstrated that protein level of PI3K p85 in H9c2 cells was decreased significantly in H/R+NC of Agomir-503 group, and it was also decreased markedly by Agomir-503 treatment under both normoxia and H/R condition (Figure 6(a)). The downstream proteins of PI3K were also detected *in vitro*. The results showed that both Akt1 and Akt2 were decreased significantly in the H/R+NC group and further decreased by Agomir-503 treatment under H/R condition (Figures 6(b) and 6(c)). Also, p-Akt (T450) and p-Akt (T308) were decreased significantly in the H/R+NC group compared with the Con+NC group (Figures 6(d) and 6(e)), p-Akt (T450) was further decreased by Agomir-503 treatment under both normoxia and H/R conditions, and Agomir-503 also tended to further decrease p-Akt (T308) under H/R condition, but the difference did not reach statistical significance (Figure 6(e)). In addition, both Stat3 and p-Stat3 (Y705) were decreased significantly in the H/R+NC group in comparison with the Con+NC group and further decreased by Agomir-503 treatment under H/R condition (Figures 6(f) and 6(g)). These results indicated that miR-503 may modulate myocardial I/R injury by blocking the prosurvival PI3K/Akt and STAT3 signaling pathways.

### 3.4. Inhibition of miR-503 Alleviated Cell Injury Induced by H/R

**Figure 7:**
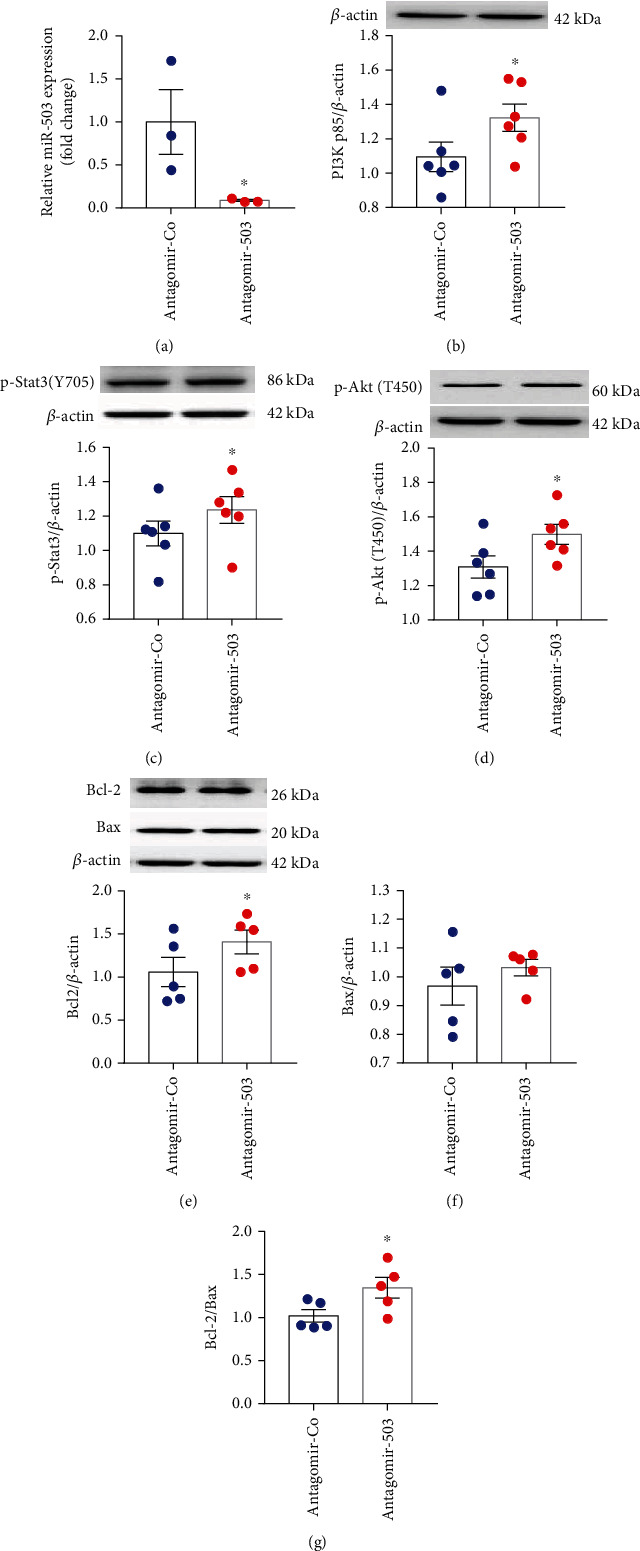
Inhibition of miR-503 in H9c2 cells under normoxia. (a) miR-503 expression level, quantified by RT-qPCR. (b–g) Western blotting analysis of PI3K p85, p-Stat3 (Y705), p-Akt (T450), Bcl-2, and Bax and densitometry analysis of proteins. Data are expressed as mean ± SEM, *n* = 6/group. ^∗^*P* < 0.05 vs. Antagomir-Co. Student's *t*-test was applied. Antgomir-Co: negative control of Antagomir-503.

**Figure 8:**
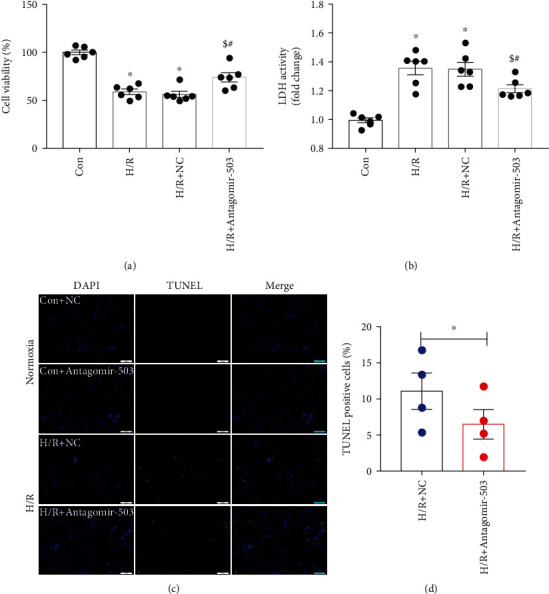
Inhibition of miR-503 in H9c2 cells alleviated cell injury under H/R condition. H9c2 cells were subjected to H/R stimulation after transfection of Antagomir-503 or NC. (a) Cell viability. (b) LDH activity. (c) Representative TUNEL staining images under both normoxia and H/R condition. Scale bar = 100 *μ*m; (d) Quantification of TUNEL-positive cells by ImageJ software. Data are expressed as mean ± SEM. (a, b) *n* = 6/group, ^∗^*P* < 0.05 vs. Con, ^$^*P* < 0.05 vs. H/R, ^#^*P* < 0.05 vs. H/R+NC. One-way ANOVA followed by Newman-Keuls multiple comparison test was applied. (d) *n* = 4/group, ^∗^*P* < 0.05 vs. H/R+NC. Student's *t*-test was applied. NC: negative control of Antagomir-503.

**Figure 9:**
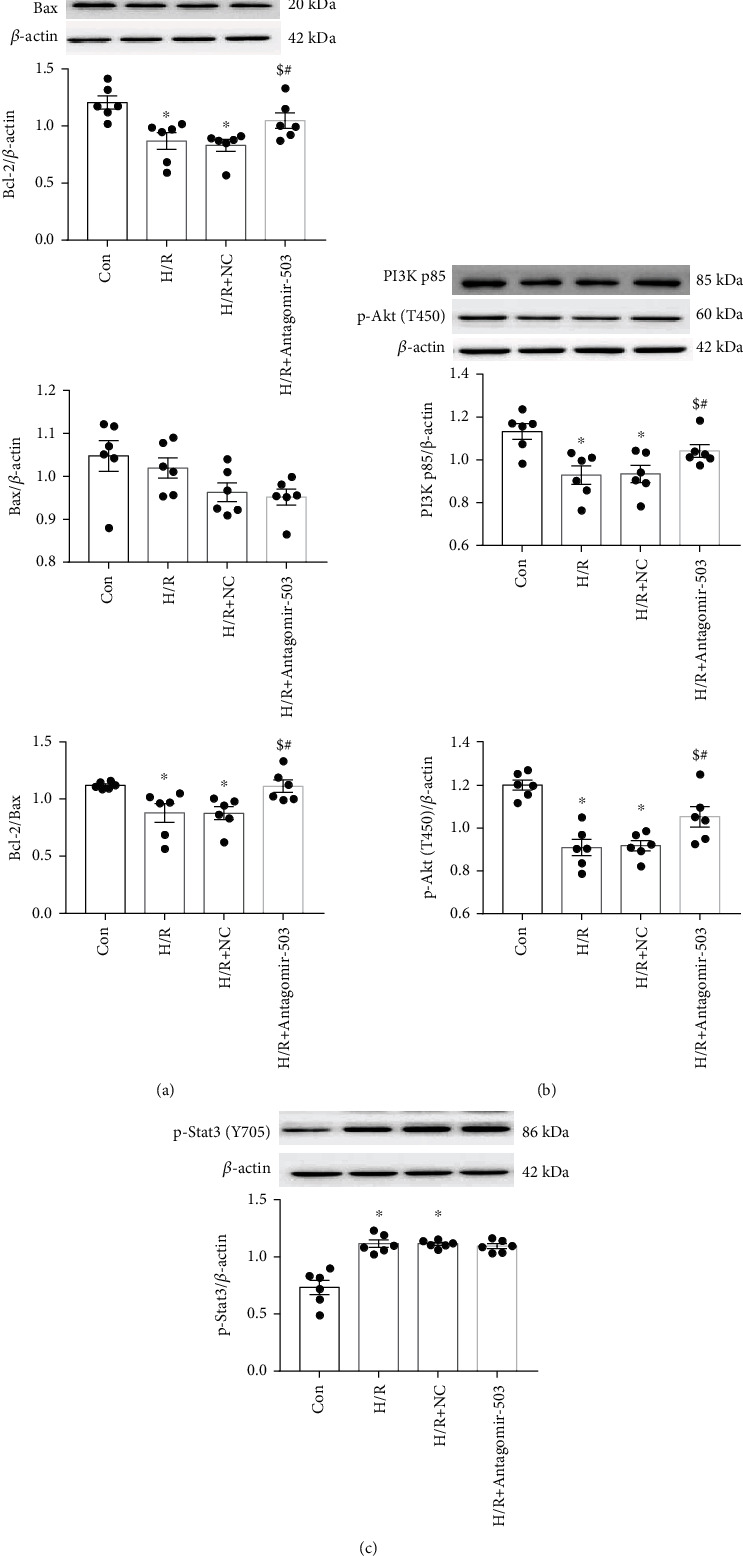
Effects of inhibiting miR-503 on protein levels involved in the PI3K/Akt and STAT3 pathways under H/R condition. (a–c)Western blotting analysis of Bcl-2, Bax, PI3K p85, p-Akt (T450), and p-Stat3 (Y705) and densitometry analysis of proteins. Data are expressed as mean ± SEM, *n* = 6/group. (a–c) ^∗^*P* < 0.05 vs. Con; ^$^*P* < 0.05 vs. H/R; ^#^*P* < 0.05 vs. H/R+NC. One-way ANOVA followed by Newman-Keuls multiple comparison test was applied.

Given that miR-503 overexpression exacerbated cell injury under H/R condition, we then investigated whether inhibition of miR-503 would alleviate cell injury induced by H/R. Antagomir-503 was applied to inhibit miR-503, and RT-qPCR analysis showed that Antagomir-503 treatment effectively inhibited miR-503 expression (Figure 7(a)). Next, the related proteins in H9c2 cells under normoxia were detected first. It was found that PI3K p85, p-Akt (T450), p-Stat3 (Y705), and Bcl-2 protein levels were increased significantly in the Antagomir-503 group compared with Antagomir-Co (negative control of Antagomir-503), while Antagomir-503 did not have significant impact on Bax (Figures 7(b)–7(g)). Therefore, we further explored whether inhibition of miR-503 under H/R condition would alleviate cell injury. The results showed that Antagomir-503 treatment increased cell viability (Figure 8(a)) and decreased LDH activity under H/R condition (Figure 8(b)). Cell apoptosis was detected by TUNEL assay, and Antagomir-503 did not have obvious impact on apoptotic cell death under normoxia (Figure 8(c)). However, inhibition of miR-503 with Antagomir-503 significantly reduced the percentage of TUNEL-positive cells under H/R condition (Figures 8(c) and 8(d)). Furthermore, Western blotting analysis showed that there was no significant difference for Bax among groups, but Bcl-2 was increased significantly by Antagomir-503 treatment under H/R condition, and the ratio of Bcl-2 to Bax was higher in H/R+Antagomir-503 group compared with H/R or H/R+NC of the Antagomir-503 group (Figure 9(a)). Under H/R condition, both PI3K p85 and p-Akt (T-450) protein levels were increased significantly by Antagomir-503 treatment (Figure 9(a)). For p-Stat3 (Y705), it was increased significantly in the H/R or H/R+NC group compared with Con, and no significance was detected in p-Stat (Y705) between the H/R+Antagomir-503 and H/R+NC groups (Figure 9(c)), but Antagomir-503 treatment increased p-Stat3 (Y705) under normoxia (Figure 8(c)). For the upstream molecule of Stat3, JAK2, and p-JAK2 (Y1007/1008), there is no significance between Con and H/R (Supplemental Figure 1). Taken together, the data indicated that inhibition of miR-503 *in vitro* activated PI3K/Akt and STAT3 signaling pathways and exerted cardioprotective effects during H/R injury.

## 4. Discussion

In the present study, it is demonstrated that miR-503 expression level was downregulated in mouse heart tissue following myocardial I/R injury *in vivo*. Consistently, it was also downregulated in H9c2 cells under both hypoxia and H/R conditions. Thus, it is indicated that miR-503 was involved in the pathogenesis of myocardial I/R injury. Further study revealed that upregulation of miR-503 aggravated cardiomyocyte injury upon H/R stimulation, while the inhibition of miR-503 alleviated cell injury. Moreover, results showed that Agomir-503 treatment in H9c2 cells resulted in reduced phosphorylation of Akt (p-Akt, T450) protein level under both normoxia and H/R condition and reduced p-Stat3 (Y705) under H/R condition, but Agomir-503 treatment had no obvious effect on p-Akt (T308) under both normoxia and H/R conditions. In contrary, Antagomir-503 treatment activated Akt (T450) under both conditions and increased Stat3 under normoxia, suggesting that miR-503 regulated myocardial I/R injury by PI3K/Akt and STAT3 signaling pathways.

Dysregulation of miR-503 exists in different diseases, such as diabetes mellitus [25], pulmonary arterial hypertension [26], and cancer [27, 28, 37, 38]. Consistent with previous studies showing that miR-503 was downregulated in rat heart following I/R and in endothelial progenitor cells under hypoxia condition [29, 39], we also found that miR-503 was downregulated in mouse heart following I/R and in H9c2 cells under hypoxia condition. However, Zhu et al. reported that miR-503 was upregulated in H9c2 cells exposed to hypoxia without oxygenation. In their experiment, cells were cultured in low glucose medium and then exposed to hypoxia (1% O_2_) at different time points [40], while in our experiment, cells were deprived of glucose and serum, and exposed to hypoxia (<1% O_2_) for 10 h or followed by 6 h of reoxygenation. This inconsistence may be caused by different culture conditions.

Most of previous studies showed that overexpression of miR-503 *in vitro* inhibited cell proliferation and increased cell apoptosis. Hirakawa et al. [41] showed that overexpression of miR-503 in human endometriotic cyst stromal cells inhibited cell proliferation and induced cell apoptosis by downregulation of its targets cyclinD1, Bcl-2, Ras homology A (RhoA), Rho-associated coiled-coil-forming protein kinase (ROCK1), ROCK2, and vascular endothelial growth factor A. Min et al. showed that miR-503 induced the apoptosis of dendritic cells *in vivo* and *in* vitro by directly targeting Bcl-2 [42]. Guo et al. [43] reported that forced expression of miR-503 reduced the cell viability of neonatal mouse ventricle cells and induced cell apoptosis under H/R condition, while silencing of miR-503 recovered cell viability and alleviated cell apoptosis. Consistent with these studies, in our study, overexpression of miR-503 in H9c2 cells reduced cell viability under either normoxia or H/R condition and increased cell apoptosis under H/R condition, while inhibition of miR-503 reversed these effects under H/R condition. The apoptotic pathway includes extrinsic pathway, intrinsic pathway, and perforin/granzyme pathway [44]. The Bcl-2 family is involved in the intrinsic pathway of apoptosis; it includes proapoptotic proteins (such as Bad, Bax or Bid) and antiapoptotic proteins (such as Bcl-2 and Bcl-XL). Bcl-2 is regulated by several signaling pathways and substances, including JAK2/STAT3, PI3K/AKT, MEK1/ERK1/2, PTEN, cardiac ankyrin repeat protein, eNOS, and miRs [45]. Bcl2 is the target gene of miR-503 [41, 42]. In our study, under normoxia condition, Bcl-2 was significantly downregulated in H9c2 cells in the presence of miR-503 overexpression, while the percentage of TUNEL-positive cells trended to increase, but the difference of TUNEL-positive cells between the Con+NC and Con+Agomir-503 group did not reach statistical significance. It is possible that other prosurvival signaling pathways may play a compensatory role under normoxia, which merits further in deep studies. Collectively, these studies demonstrated that overexpression of miR-503 contributed to the development of myocardial I/R injury.

Although the present study demonstrated that miR-503 was downregulated in myocardial I/R injury, the underlying mechanism is unknown. We have analyzed the mechanism of abnormal expression of miR-503 in our latest review [46]. The reasons of miR-503 downregulation have been investigated by several studies. Zhou et al. [28] and Hirakawa et al. [41] have demonstrated that miR-503 downregulation in tissues of hepatocellular carcinomas or human endometriotic cyst stromal cells may be caused by DNA hypermethylation near the promoter of miR-503. Yan et al. [47] reported that long noncoding RNA (lncRNA) MALAT1 (metastasis-associated lung adenocarcinoma transcript 1) downregulated miR-503 because of bonding to miR-503 as a sponge. It is reported that MALAT1 is increased in peripheral blood cells of patients with acute myocardial infarction [48] or heart tissues of rat model of myocardial I/R injury [49]. The downregulation of miR-503 in myocardial I/R injury may be caused by epigenetic modulation of the promoter of miR-503 or upregulation of lncRNA MALAT1; however, this hypothesis needs further study.

miRs regulate their target genes by mRNA degradation or translation suppression. PIK3R1 and BCL2 are the predicted target genes of miR-503. PIK3R1, also known as PI3K p85, encodes the 85 kDa regulatory subunit of Class I PI3K [10]. The luciferase assay for PI3K p85 and BCL2 was omitted, as it has already performed by others previously [27, 42, 47]. In the present study, Western blotting analysis has demonstrated that the protein levels of both PI3K p85 and Bcl-2 were decreased significantly by Agomir-503 treatment under both normoxia and H/R condition and increased by Antagomir-503 treatment *in vitro*. One miRNA can target multiple genes simultaneously, and the same gene can be targeted by multiple miRNAs cooperatively. Other studies have demonstrated that PI3K p85 is the miR-21 target gene [50], and Bcl-2 is the miR-34a target gene [51]. Therefore, during myocardial I/R injury, PI3K P85 and Bcl-2 may also be regulated by other miRs simultaneously. Stat3 is not the predicted target gene of miR-503, while overexpression of miR-503 reduced the protein expression of Stat3 and p-Stat3 (Y705) under H/R condition, suggesting that Stat3 is at least an indirect target gene of miR-503, which is similar in nature as reported that Stat3 is the target gene of other miRs like miR-125a and miR-17 [52, 53]. The exact relationship between Stat3 and miR-503 is unknown in myocardial I/R injury, and it will be our future study. Taken together, multiple miRs may work cooperatively to regulate their targets and form the complex miR regulatory network. Exploring this complex network is indispensable for finding novel therapeutic targets for myocardial I/R injury.

PI3K p85, as the target gene of miR-503, was decreased significantly in mouse heart following I/R *in vivo*, and the downstream molecules p-Akt (T450) and p-Akt (T308) were also decreased significantly *in vivo*. Consistent with previous study, Hao et al. reported that protein PI3K, Akt, and p-Akt were all decreased in the rat heart tissues following I/R [54], while authors did not show the phosphorylated residue of Akt clearly. *In vitro*, PI3K p85, Akt1, Akt2, p-Akt (T450), and p-Akt (T308) protein levels were downregulated under H/R condition, and overexpression of miR-503 reduced both PI3K p85 and p-Akt (T450) protein levels. Yan et al. [47] reported that overexpression of miR-503 reduced p-Akt (S473) protein level in mouse lung tissue of the pulmonary fibrosis model. In our study, p-Akt (T308) protein level was decreased *in vivo*, while overexpression of miR-503 did not further reduce the p-Akt (T308) protein level *in vitro*; the mechanism is complicated and needs further study.

The JAK/STAT3 signaling pathway is one of important prosurvival signaling pathways in myocardial I/R injury, while JAK and STAT3 are not the target genes of miR-503. As the upstream molecule of Stat3, JAK2, and p-JAK2, no significance was found between Con and H/R (Supplemental Figure 1); thus, we did not explore it further in this study. *In vivo*, p-Stat3 (Y705) was increased significantly in the I/R group, while PI3K p85 and p-Akt (T450 and T308) were decreased markedly; it is indicative that the PI3K/Akt and STAT3 pathways may not change in parallel for cardioprotection in this mouse model. *In vitro*, we found that Agomir-503 treatment reduced p-Stat3 (Y705) protein level in H/R+NC of the Agomir-503 group compared with the Con+NC group, inhibition of miR-503 with Antagomir-503 increased p-Stat3 (Y705) under normoxia, but under H/R condition, no significant difference was detected in p-Stat3 between the H/R+NC and H/R+Antagomir-503 groups (Figure 9(c)). It is inferred that the increase of p-Stat3 under H/R condition may play a compensatory role of cardioprotection. At the time point of hypoxia 10 h and rexoygenation 6 h, the increase of p-Stat3 may have reached the plateau; it is difficult to further increase p-Stat3 expression level with inhibition of miR-503. Previous studies showed that Akt can be activated at low and high cell densities *in vitro*, but Stat3 was not activated at low cell density; it can be activated at high cell density or cell aggregation by cadherin engagement [55, 56]. Therefore, Akt and Stat3 activations are not parallel at certain condition, and cadherin engagement may be one of mechanisms to explain this difference. Taken together, these findings suggested that both PI3K/Akt and STAT3 signaling pathways were regulated by miR-503 during myocardial I/R injury.

Some limitations of the present study should be emphasized. Firstly, Akt has three family members; we did not detect Akt3 in the present study. Although Akt3 has lower expression in the heart, it may still play an important role in cardiomyocyte proliferation and apoptosis [57]. Secondly, overexpression or inhibition of miR-503 *in vivo* could better reveal the potential therapeutic effects of miR-503; thus, related experiments should be performed in future studies. Thirdly, the H9c2 cell line is originated from the heart of rat species, the HL-1 cell line is from mouse atrial cardiomyocyte tumor lineage, and the animals we used are C57BL/6N mice. This is the potential limitation in our study. Rat and mouse cell lines have similarities in the aspect we studied, despite that their metabolism being a bit different, but in the current study, we did not study metabolism. It will be better to match the animal species in vivo experiment and cell line-derived species in future studies. Finally, during myocardial ischemia/reperfusion injury, there are several kinds of regulated cell death, such as apoptosis, necrosis, autophagy-dependent cell death, ferroptosis, pyroptosis, and parthanatos [58]. Apoptosis is part of our current study, other kinds of regulated cell death need to be investigated in the future.

In conclusion, the results demonstrated that miR-503 was downregulated in myocardial I/R injury, and its downregulation may be one compensatory protection mechanism in the pathological process of myocardial I/R injury; it regulated myocardial I/R injury via the PI3K/Akt and STAT3 pathways. These findings indicated that miR-503 may become a therapeutic target in preventing myocardial I/R injury.

## Data Availability

The data used to support the findings of this study is available from the corresponding author upon request.

## Supplementary Materials

Supplementary Materials Supplemental Figure 1: the protein expression of JAK2 and p-JAK2 (Y1007/1008) in H9c2 cells upon H/R stimulation. Western blotting analysis of JAK2 and p-JAK2 and densitometry analysis of proteins. Data are expressed as mean ± SEM, *n* = 6/group. Student's *t*-test was applied.
