# Supplementary figures and images for: MicroRNA-503 Exacerbates Myocardial Ischemia/Reperfusion Injury via Inhibiting PI3K/Akt- and STAT3-Dependent Prosurvival Signaling Pathways
Source: Oxid Med Cell Longev. 2022 May 17;2022:3449739. doi: 10.1155/2022/3449739 (PMC9130001; doi:10.1155/2022/3449739)

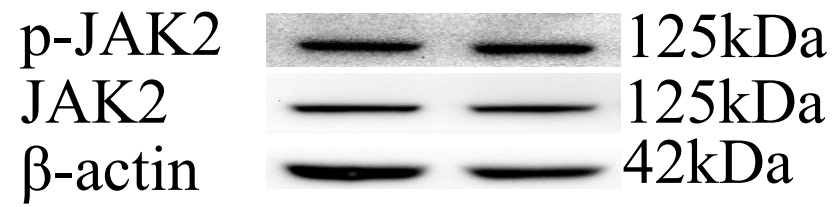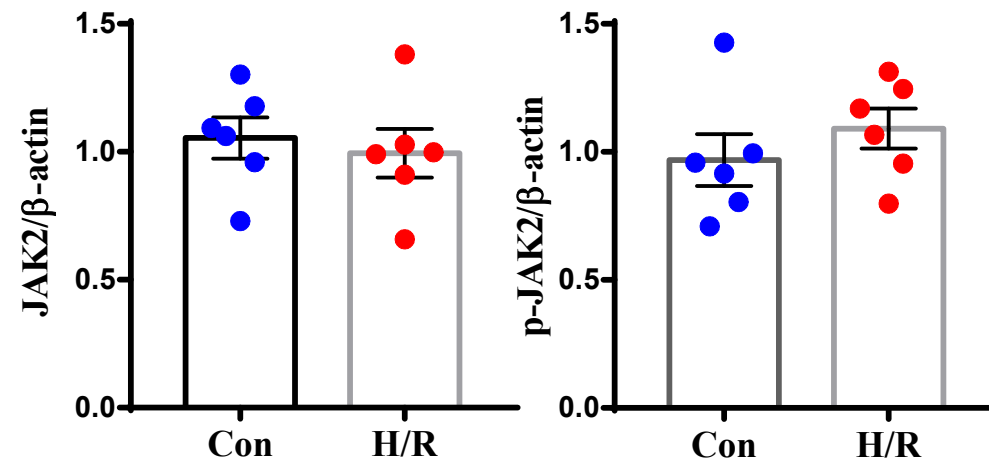

Supplement: Supplementary Materials — Supplemental Figure 1: the protein expression of JAK2 and p-JAK2 (Y1007/1008) in H9c2 cells upon H/R stimulation. Western blotting analysis of JAK2 and p-JAK2 and densitometry analysis of proteins. Data are expressed as mean ± SEM, n = 6/group. Student's t-test was applied. [file 3449739.f1.pdf]
